# Antidepressant- and Anxiolytic-Like Effects of New Dual 5-HT_1A_ and 5-HT_7_ Antagonists in Animal Models

**DOI:** 10.1371/journal.pone.0142499

**Published:** 2015-11-10

**Authors:** Karolina Pytka, Anna Partyka, Magdalena Jastrzębska-Więsek, Agata Siwek, Monika Głuch-Lutwin, Barbara Mordyl, Grzegorz Kazek, Anna Rapacz, Adrian Olczyk, Adam Gałuszka, Marian Błachuta, Anna Waszkielewicz, Henryk Marona, Jacek Sapa, Barbara Filipek, Anna Wesołowska

**Affiliations:** 1 Department of Pharmacodynamics, Faculty of Pharmacy, Jagiellonian University Medical College, Medyczna 9, 30–688, Krakow, Poland; 2 Department of Clinical Pharmacy, Faculty of Pharmacy, Jagiellonian University Medical College, Medyczna 9, 30–688, Krakow, Poland; 3 Department of Pharmacobiology, Faculty of Pharmacy, Jagiellonian University Medical College, Medyczna 9, 30–688, Krakow, Poland; 4 Institute of Automatic Control, Silesian University of Technology, Akademicka 16, 44–100, Gliwice, Poland; 5 Department of Bioorganic Chemistry, Chair of Organic Chemistry, Faculty of Pharmacy, Jagiellonian University Medical College, Medyczna 9, 30–688, Krakow, Poland; Radboud University Medical Centre, NETHERLANDS

## Abstract

The aim of this study was to further characterize pharmacological properties of two phenylpiperazine derivatives: 1-{2-[2-(2,6-dimethlphenoxy)ethoxy]ethyl}-4-(2-methoxyphenyl)piperazynine hydrochloride (HBK-14) and 2-[2-(2-chloro-6-methylphenoxy)ethoxy]ethyl-4-(2- methoxyphenyl)piperazynine dihydrochloride (HBK-15) in radioligand binding and functional *in vitro* assays as well as *in vivo* models. Antidepressant-like properties were investigated in the forced swim test (FST) in mice and rats. Anxiolytic-like activity was evaluated in the four-plate test in mice and elevated plus maze test (EPM) in rats. Imipramine and escitalopram were used as reference drugs in the FST, and diazepam was used as a standard anxiolytic drug in animal models of anxiety. Our results indicate that HBK-14 and HBK-15 possess high or moderate affinity for serotonergic 5-HT_2_, adrenergic α_1_, and dopaminergic D_2_ receptors as well as being full 5-HT_1A_ and 5-HT_7_ receptor antagonists. We also present their potent antidepressant-like activity (HBK-14—FST mice: 2.5 and 5 mg/kg; FST rats: 5 mg/kg) and (HBK-15—FST mice: 1.25, 2.5 and 5 mg/kg; FST rats: 1.25 and 2.5 mg/kg). We show that HBK-14 (four-plate test: 2.5 and 5 mg/kg; EPM: 2.5 mg/kg) and HBK-15 (four-plate test: 2.5 and 5 mg/kg; EPM: 5 mg/kg) possess anxiolytic-like properties. Among the two, HBK-15 has stronger antidepressant-like properties, and HBK-14 displays greater anxiolytic-like activity. Lastly, we demonstrate the involvement of serotonergic system, particularly 5-HT_1A_ receptor, in the antidepressant- and anxiolytic-like actions of investigated compounds.

## Introduction

Depression is a very serious mood disorder, characterized by low mood, anhedonia, reduced energy, and often comorbid with anxiety. Its unclear aetiology may involve genetic factors, abnormal neurotransmission in the central nervous system (CNS), neuroendocrine or immunological processes. Patients suffering from depressive disorders have impaired serotonergic 5-HT_1A_ receptor function [1–3]. Post-mortem studies demonstrated some alterations in agonist-stimulated 5-HT_1A_ receptor activation in depressed suicide victims [4]. Furthermore, a C(-1019)G (rs6295) promoter polymorphism of the 5-HT_1A_ receptor gene (HTR1A) has been identified and has been proven to increase the risk of affective disorders and the resistance to selective serotonin reuptake inhibitors (SSRIs) treatment [5]. Serotonergic 5-HT_1A_ receptors were found in many brain regions, including limbic structures and cerebral cortex, and are involved in many physiological and pathological processes [6]. It is not surprising though that many studies on 5-HT_1A_ receptor ligands with the possible use in the treatment of mood disorders are still being performed. Some of 5-HT_1A_ receptor ligands are already used in the therapy of depression, e.g. vilazodone and vortioxetine (serotonin reuptake inhibitors and partial 5-HT_1A_ agonists) or generalized anxiety disorder–buspirone (a partial 5-HT_1A_ receptor agonist) [7,8]. 5-HT_1A_ antagonists haven’t been introduced to the treatment of depression so far but they may have beneficial effects i.e. accelerating/enhancing the clinical effects of SSRIs [9]. This could be achieved by preventing 5-HT_1A_- autoreceptor-mediated negative feedback. Pindolol, a non-selective β-adrenoceptor and 5-HT_1A_ receptor antagonist, enhanced the efficacy of SSRIs in depressed patients [10]; however, its effect on cardio-vascular system limits its clinical use. Another compound DU-125530—a potent pre- and postsynaptic 5-HT_1A_ receptor antagonist—augmented SSRI-induced increases in extracellular 5-HT but did not accelerate the effects of fluoxetine in depressed individuals [11]. The authors suggested that the blockade of postsynaptic 5-HT_1A_ receptors canceled the benefits of enhancing presynaptic 5-hydroxytryptaminergic function. Therefore, we should search for selective presynaptic 5-HT_1A_ antagonists or multimodal compounds with weak 5-HT_1A_ antagonistic properties.

Analogously, there is evidence that 5-HT_7_ receptors play an important role in affective disorders [12,13]. Although the detailed analysis of 5-HT_7_ receptor distribution in individuals with depression or anxiety is not available yet, recent animal studies showed up-regulation of 5-HT_7_ receptors in the hippocampus after exposure to stress [14,15], and these adaptive changes were inhibited by fluoxetine [14,15]. Interestingly, 5-HT_7_ knockout mice exhibit a behavioural phenotype similar to mice treated with antidepressants [16,17]. Moreover, a selective 5-HT_7_ receptor antagonist SB 269970 produced antidepressant- and anxiolytic-like effects in behavioural animal models. It is also worth mentioning that vortioxetine is a 5-HT_7_ receptor antagonist.

Literature data indicate that phenylpiperazine derivatives present various potential therapeutic properties, including analgesic, antipsychotic, antidepressant and/or anxiolytic detected in animal models, thus this group of compounds has been widely studied by many researchers [18–25].

In the present study antidepressant- and anxiolytic-like activity, as well as a possible mechanism of action of two 2-methoxyphenylpiperazine derivatives: 1-{2-[2-(2,6-dimethlphenoxy)ethoxy]ethyl}-4-(2-methoxyphenyl)piperazynine hydrochloride (HBK-14) and 2-[2-(2-chloro-6-methylphenoxy)ethoxy]ethyl-4-(2- methoxyphenyl)piperazynine dihydrochloride (HBK-15) were evaluated in preclinical models sensitive to clinically effective antidepressants and anxiolytics in mice and rats. Since in our previous studies HBK-14 and HBK-15 showed high or moderate affinity for 5-HT_1A_ (K_i_ = 41 nM, K_i_<1 nM, respectively) and 5-HT_7_ receptors (K_i_ = 156 nM, K_i_ = 34 nM, respectively), the aim of this study was to determine their intrinsic activity towards the above receptors as well as to broaden their affinity profile *in vitro*. As monoaminergic receptors are known to play a pivotal role in antidepressant and anxiolytic activity, behavioural studies were conducted to establish the potential of these compounds for such actions.

## Methods

### Animals

Experiments were performed on adult male mice (CD-1, 18–21 g) or male rats (Wistar, 170–220 g), purchased from Animal Facility at the Faculty of Pharmacy, Jagiellonian University Medical College, Krakow, Poland. Animals were kept in standard cages at room temperature of 22 ± 2°C under light/dark (12:12) cycle, and had free access to food (standard laboratory pellets) and water before experiments. All experiments were performed between 9 a.m. and 2 p.m. For all experiments animals were selected randomly. The animals were used only once in each test. All injections were given in a volume of 10 ml/kg (mice) and 2 ml/kg (rats). Each experimental group consisted of 10 (mice) or 8 (rats) animals. A trained observer blind to the treatments scored behavioural experiments. All experimental procedures involving animals were conducted in accordance with European Union and Polish legislation acts concerning animal experimentation and approved by the I Local Ethics Committee for Experiments on Animals of the Jagiellonian University in Krakow, Poland (approval numbers: 74/2012, 7/2013, 52/2014 and 114/2015). All efforts were made to minimize suffering and to reduce the number of animals used in the experiments.

### Drugs

**Fig 1:**
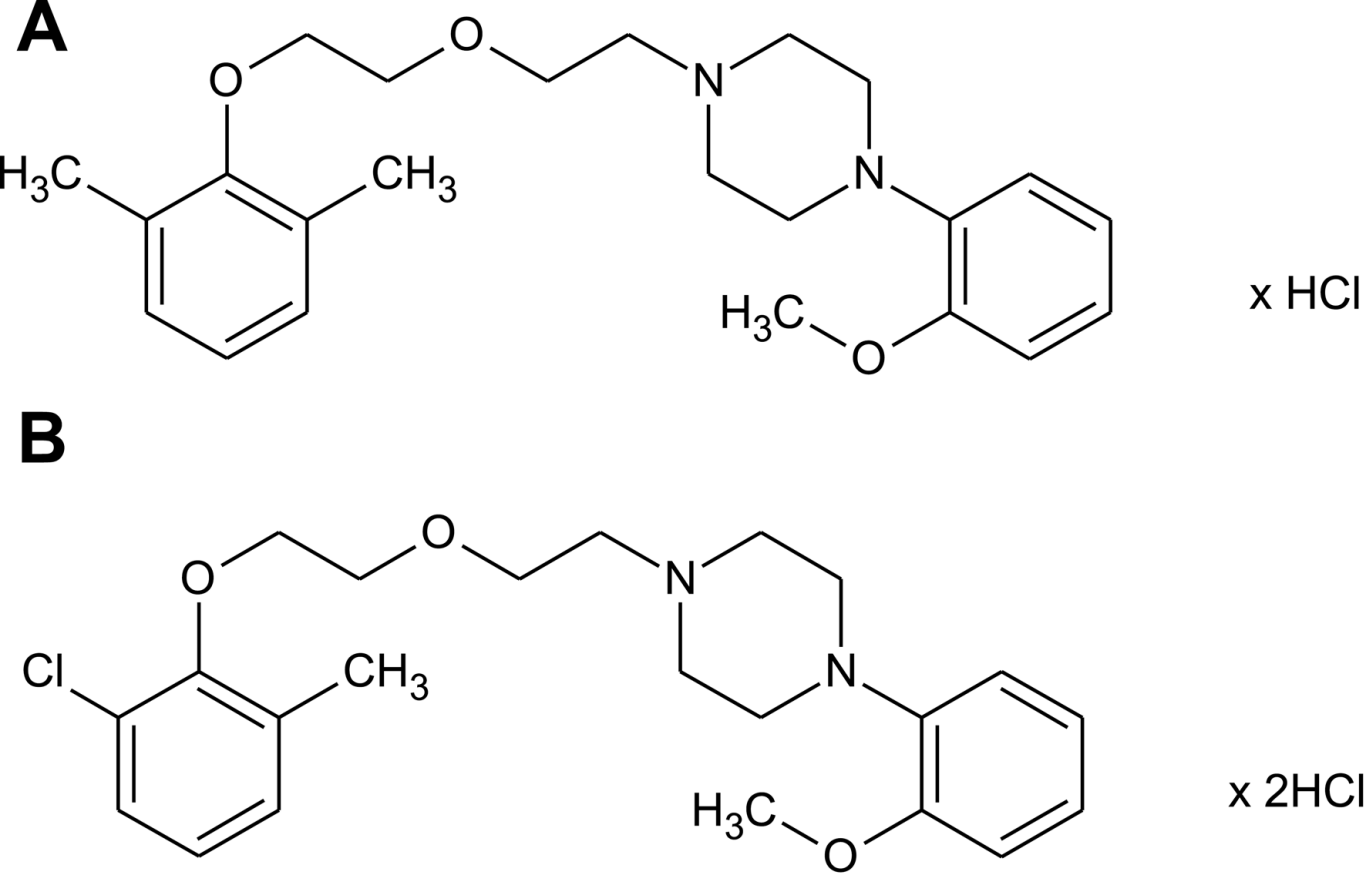
Chemical structures of HBK-14 and HBK-15. (A) 1-{2-[2-(2,6-dimethlphenoxy)ethoxy]ethyl}-4-(2-methoxyphenyl)piperazynine hydrochloride—HBK-14, (B) 2-[2-(2-chloro-6-methylphenoxy)ethoxy]ethyl-4-(2- methoxyphenyl)piperazynine dihydrochloride—HBK-15

Two studied phenylpiperazine derivatives shown in Fig 1: 1-{2-[2-(2,6-dimethlphenoxy)ethoxy]ethyl}-4-(2-methoxyphenyl)piperazynine hydrochloride (HBK-14) and 2-[2-(2-chloro-6-methylphenoxy)ethoxy]ethyl-4-(2- methoxyphenyl)piperazynine dihydrochloride (HBK-15) were synthesized in the Department of Bioorganic Chemistry, Chair of Organic Chemistry, Pharmacy Faculty, Jagiellonian University, Krakow, Poland [26]. HBK-14, HBK-15, diazepam (Tocris, United Kingdom), imipramine (Sigma-Aldrich, Germany), escitalopram (Lundbeck, Denmark), fluoxetine (Sigma-Aldrich, Germany), reboxetine (Sigma-Aldrich, Germany), and bupropion (Sigma-Aldrich, Germany) were dissolved in saline and administered intraperitoneally (i.p.) 30 min before each test. p-Chlorophenylalanine (pCPA) was suspended in 1% Tween 80 solution and administered i.p. for 3 consecutive days. N-{2-[4-(2-metho xyphenyl)-1-piperazinyl] ethyl}- N-(2-pyridinyl)cyclohexanecarboxamide trihydrochloride (WAY-100635) was dissolved in saline and injected subcutaneously, 15 min before the studied compounds. The control groups received 0.9% NaCl (i.p.). For radioligand and functional experiments mianserin (Sigma-Aldrich, Germany), escitalopram (Lundbeck, Denmark), phentolamine (Sigma-Aldrich, Germany), clonidine (Sigma-Aldrich, Germany), propranolol (Sigma-Aldrich, Germany), haloperidol (Sigma-Aldrich, Germany), serotonin (Sigma-Aldrich, Germany), and SB 269970 (Tocris, United Kingdom) were dissolved in dimethyl sulfoxide (DMSO) and used as reference compounds.

### Radioligand binding assays

#### Serotonergic 5-HT_2A_ receptor

Radioligand binding was performed using membranes from CHO-K1 cells stably transfected with the human 5-HT_2A_ receptor. Monolayer of CHO-K1 cells with stable expression of human receptor after reaching about 90% confluence was washed twice with PBS. After that the cells were scrapped and collected to centrifuge tubes. About 20 volumes of assay buffer was added to each tube and cells were homogenized (3 x 10 sec., 24 000 rpm; Ultra Turrax T25B homogenizator, IKA Labortechnik, USA). Tubes were centrifuged (20 min., 30 000 x g, about 4°C; 3K30 centrifuge, Sigma Laborzentrifugen, Germany). Supernatant was discarded and appropriate volume of assay buffer with 10% glycerol was added and homogenization was repeated. Membrane suspension was aliquoted into cryotubes, freezed in liquid nitrogen and stored until assay in -80°C. Membrane protein concentration was determined by BCA method. Immediately before assay frozen membranes were thawed quickly (about 35°C), mixed and homogenized (60 sec., 24,000 rpm) with appropriate volume of assay buffer to reach desired membrane protein concentration. All assays were carried out in duplicates. Determination of the equilibrium dissociation constant (K_D_) was conducted by saturation experiments with increasing series of concentrations of a [3H]-ketanserin (0.05–3.0 nM). 50 μl working solution of the tested compounds, 50 μl [^3^H]-ketanserin (final concentration 0,5 nM, K_D_ 0.3 nM) and 150 μl diluted membranes (5 μg protein per well) prepared in assay buffer (50 mM Tris, pH 7.4, 4 mM CaCl_2_, 0.1% ascorbic acid) were transferred to polypropylene 96-well microplate using 96-wells pipetting station Rainin Liquidator (MettlerToledo). Serotonin (100 μM) was used to define nonspecific binding. Microplate was covered with a sealing tape, mixed and incubated for 60 minutes at 27°C. The reaction was terminated by rapid filtration through GF/B filter mate presoaked with 0.3% polyethyleneimine for 30 minutes. Ten rapid washes with 200 μl 50 mM Tris buffer (4°C, pH 7.4) were performed using automated harvester system Harvester-96 MACH III FM (Tomtec). The filter mates were dried at 37°C in forced air fan incubator and then solid scintillator MeltiLex was melted on filter mates at 90°C for 5 minutes. Radioactivity was counted in MicroBeta2 scintillation counter (PerkinElmer) at approximately 30% efficiency. Inhibition constants (K_i_) were calculated.

#### Serotonin transporter (SERT)

The experiment was performed according to the slightly modified method described by Owens et al. [27]. Rat cerebral cortex was homogenized in 30 volumes of ice-cold 50 mM Tris-HCl containing 150 mM NaCl and 5 mM KCl, pH = 7.7 at 25°C and centrifuged at 20,000xg for 20 min. The supernatant was decanted and pellet was re-suspended in 30 volumes of buffer and centrifuged again. The resulting pellet was re-suspended in the same quantity of the buffer and centrifuged third time in the same conditions. [^3^H]-citalopram (spec. act. 85,6 Ci/mmol, Perkin Elmer) was used for labelling 5-HT-transporter. 240 μl of the tissue suspension, 30 μl of 1 μM imipramine (displacer), 30 μl of 1nM [^3^H]-citalopram and 30 μl of the analysed compound were incubated at 24°C for 1 h. The concentrations of analysed compounds ranged from 10^−10^ to 10^−5^ M. The incubation was terminated by rapid filtration over glass fibre filters FilterMate B (PerkinElmer, USA) using 96-well FilterMate harvester (PerkinElmer, USA). K_i_ values were calculated.

#### α_1_-, α_2_- and β_1_-adrenoceptors

The experiments were performed using rat cerebral cortex. [^3^H]-prazosin (19.5 Ci/mmol, α_1_-adrenoceptor), [^3^H]-clonidine (70.5 Ci/mmol, α_2_-adrenoceptor) and [^3^H]-CGP-12177 (48 Ci/mmol, β_1_-adrenoceptor) were used. The membrane preparation and the assay procedures were conducted according to the slightly modified method described by Maj et al. [28]. Rat brains were homogenized in 20 volumes of ice-cold 50 mM Tris–HCl buffer (pH 7.6) and centrifuged at 20,000xg for 20 min (0–4°C). The cell pellet was re-suspended in Tris–HCl buffer and centrifuged again. The final incubation mixture (final volume 300 μL) consisted of 240 μL membrane suspension, 30 μL of a [^3^H]-prazosin (0.2 nM), [^3^H]-clonidine (2 nM) or [^3^H]-CGP-12177 (0.2 nM) solution and 30 μL buffer containing from seven to eight concentrations (10^−11^–10^−4^ M) of investigated compounds. To measure unspecific binding, phentolamine -10 μM (for [^3^H]-prazosin), 10 μM clonidine (for [^3^H]-clonidine) and propranolol -1 μM (for [^3^H]-CGP-12177), were applied. Radioactivity was measured in a WALLAC 1409 DSA liquid scintillation counter (Perkin Elmer, USA). All assays were done in duplicates. K_i_ values were calculated.

#### Dopaminergic D_2_ receptor

Radioligand binding was performed using membranes from CHO-K1 cells stably transfected with the human D_2_ receptor. All assays were carried out in duplicates. K_D_ for receptor radioligand was determined by saturation experiments with increasing series of concentrations of [^3^H]-methylspiperon (0.05–3.0 nM). 50 μl working solution of the tested compounds, 50 μl [^3^H]-methylspiperon (final concentration 0.4 nM, K_D_ 0.4 nM) and 150 μl diluted membranes (10 μg protein per well) prepared in assay buffer (50 mM HEPES, pH 7.4, 50 mM NaCl, 5 mM MgCl_2_, 0.5 mM EDTA) were transferred to polypropylene 96-well microplate using 96-wells pipetting station Rainin Liquidator (MettlerToledo). (+)-butaclamol (10 μM) was used to define nonspecific binding. Microplate was covered with a sealing tape, mixed and incubated for 60 minutes at 37°C. The reaction was terminated by rapid filtration through GF/C filter mate pre-soaked with 0.3% polyethyleneimine for 30 minutes. Ten rapid washes with 200 μl 50 mM Tris buffer (4°C, pH 7.4) were performed using automated harvester system Harvester-96 MACH III FM (Tomtec). The filter mates were dried at 37°C in forced air fan incubator and then solid scintillator MeltiLex was melted on filter mates at 90°C for 5 minutes. Radioactivity was counted in MicroBeta2 scintillation counter (PerkinElmer) at approximately 30% efficiency. K_i_ values were calculated.

### Functional assays

#### 5-HT_1A_ receptor

Test and reference compounds were dissolved in DMSO at a concentration of 1 mM. Serial dilutions were prepared in 96-well microplate in assay buffer and 8 to 10 concentrations were tested. A cellular aequorin-based functional assay was performed with recombinant CHO-K1 cells expressing mitochondrially targeted aequorin, human GPCR and the promiscuous G protein α16 for 5-HT_1A_. Assay was executed according to previously described protocol [29]. After thawing, cells were transferred to assay buffer (DMEM/HAM’s F12 with 0.1% protease free BSA) and centrifuged. The cell pellet was resuspended in assay buffer and coelenterazine h was added at final concentrations of 5 μM. The cells suspension was incubated at 16°C, protected from light with constant agitation for 16 h and then diluted with assay buffer to the concentration of 100,000 cells/ml. After 1 h of incubation, 50 μl of the cells suspension was dispensed using automatic injectors built into the radiometric and luminescence plate counter MicroBeta2 LumiJET (PerkinElmer, USA) into white opaque 96-well microplates preloaded with test compounds. Immediate light emission generated following calcium mobilization was recorded for 30 s. In antagonist mode, after 25 min of incubation the reference agonist was added to the above assay mix and light emission was recorded again. Final concentration of the reference agonist was equal to EC_80_ (100 nM serotonin). IC_50_ and EC_50_ values were calculated.

#### 5-HT_7_ receptor

Test and reference compounds were dissolved in DMSO at a concentration of 1 mM. Serial dilutions were prepared in 96-well microplate in assay buffer and 8 to 10 concentrations were tested. For the 5-HT_7_ receptor, adenylyl cyclase activity was monitored using cryopreserved CHO-K1 cells with expression of the human serotonin 5-HT_7_ receptor. Functional assay based on cells with expression of the human 5-HT_7_ receptor was performed, according to the previously described protocol [29]. CHO-K1 cells were transfected with a beta lactamase (bla) reporter gene under control of the cyclic AMP response element (CRE) (Life Technologies).

Thawed cells were resuspended in stimulation buffer (HBSS, 5 mM HEPES, 0.5 IBMX, and 0.1% BSA at pH 7.4) at 2x105 cells/ml for 5-HT_7_ receptor. The same volume (10 μl) of cell suspension was added to tested compounds for 5-HT_7_ receptor. Samples were loaded onto a white opaque half area 96-well microplate. The antagonist response experiment was performed with 10 nM serotonin as the reference agonist for 5-HT_7_ receptor. The agonist and antagonist were added simultaneously. Cell stimulation was performed for 1h at room temperature. After incubation, cAMP measurements were performed with homogeneous TR-FRET immunoassay using the LANCE Ultra cAMP kit (PerkinElmer, USA). 10 μl of EucAMP Tracer Working Solution and 10 μl of ULight-anti-cAMP Tracer Working Solution were added, mixed, and incubated for 1 h. The TR-FRET signal was read on an EnVision microplate reader (PerkinElmer, USA). IC_50_ and EC_50_ values were calculated.

### Forced swim test (FST) in mice

The studies were carried out on mice according to the method described by Porsolt et al. [30]. The animals were dropped individually into glass cylinders (height 25 cm, diameter 10 cm) containing 10 cm of water, maintained at 23–25°C. Mice were left in the cylinder for 6 min. After the first 2 min of adaptation, the total duration of immobility was measured during the following 4-min test. The mouse was judged to be immobile when it remained floating passively in the water.

Studied compounds and reference drugs were administered (i.p.) 30 min before the test. The sub-effective doses of antidepressants (fluoxetine, reboxetine and bupropion) used in the experiments were selected based on a dose-response curve described in our previous studies [31]. To investigate the possible involvement of 5-HT_1A_ receptors in antidepressant-like activity of studied compounds in the FST, mice were pretreated with WAY-100635 (0.1 mg/kg, s.c.) 15 min before the injection of HBK-14 or HBK-15, and after 30 min the FST was performed. The dose of WAY-100635 used in the experiments was based on the literature data [32].

### Locomotor activity in mice

Locomotor activity of mice was measured in photoresistor actometers (Ugo Basile, Italy) connected to a counter for the recording of light-beam interruptions. Mice were placed individually in cages for 30 min and then the number of crossings of the light beams was recorded as the locomotor activity between 2^nd^ and 6^th^ min or for 60 s (i.e. time equal to the observation period in FST or the four-plate test, respectively). Studied compounds were administered (i.p.) 30 min before the test.

### FST in rats

The experiment was carried out according to the method of Detke et al. [33]. On the first experimental day rats were placed individually in Plexiglas cylinders (40 cm height, 18 cm in diameter) containing 30 cm of water maintained at 25°C for 15 min. After that time the rats were placed for 30 min under a 60-W bulb to dry. 24 hours after the pre-test, the rats were placed again in the cylinders and the total duration of immobility (when a rat remained floating in the water without struggling and was making only those movements necessary to keep its head above water), swimming (when a rat was making active swimming motions, more than necessary to merely maintain its head above water, e.g. moving around in the cylinder) and climbing (when a rat was making active movements with its forepaws in and out of the water, usually directed against the walls) was recorded during the whole 5-min test period. Fresh water was used for each animal. Studied compounds and reference drugs were administered (i.p.) 30 min before the test.

### Serotonergic depletion

In order to examine the possible involvement of a serotonergic system to the effect of HBK-14 and HBK-15 in the FST, the mice were pretreated with an inhibitor of tryptophan hydroxylase, involved in the serotonin synthesis, i.e. pCPA, according to the method described by Pytka et al. [31,34]. Once daily for 3 consecutive days the mice were injected i.p. with either pCPA at a dose of 200 mg/kg or 1% Tween 80 solution, depending on the group. On the test day, 24 h after the last pCPA administration and 30 min before the FST, mice received HBK-14, HBK-15 or 0.9% NaCl.

### Four-plate test in mice

The four-plate test was carried out according to the method described by Aron et al. [35]. The box (BIOSEB, Vitrolles, France) was made of an opaque plastic and was rectangular (25 cm x 18 cm x 16 cm) in shape. The floor was covered with four rectangular metal plates (11 cm x 8 cm), separated by a 4 mm gap. The plates were connected to a source of continuous current, which enabled a 120 V difference of potential between two adjacent plates for 0.5 s when the experimenter pressed the switch. Mice were placed individually onto the plate, and were allowed to explore for 15 s. Afterwards, each time a mouse passed from one plate to another, the experimenter electrified the whole floor which evoked a visible flight reaction of the animal. If the animal continued running, it received no new shocks for the following 3 s. The episodes of punished crossings were counted for 60 s.

Studied compounds and reference drugs were administered (i.p.) 30 min before the test. To investigate the possible involvement of 5-HT_1A_ receptors in anxiolytic-like activity of studied compounds in the four-plate test, mice were pretreated with WAY-100635 (0.1 mg/kg, s.c.) 15 min before the injection of HBK-14 or HBK-15, and after 30 min the four-plate test was performed. The dose of WAY-100635 used in the experiments was based on the literature data [32].

### Elevated plus maze (EPM) test in rats

Plus-maze apparatus (an automated device produced by Campden Instruments Ltd, UK) made of durable, high density, nonporous black plastic, elevated to a height of 50 cm, consisted of two open arms (50 cm x 10 cm) and two closed arms (50 cm x 10 cm, and 30 cm high walls), arranged so that the two arms of each type were opposite each other. Floor of the plus-maze was made of infrared transparent material what means that there are no visible sensors. The plus-maze was placed in a darkened room; only the centre of the apparatus was illuminated with low-density light (30 lux measured on the maze level). Plus-maze apparatus was connected to PC software by control chassis. Each rat was gently placed in the centre of the plus-maze, facing one of the closed arms, immediately after a 5-min adaptation period in a plastic black box (60 cm x 60 cm x 35 cm). During a 5-min test period, automated Motor Monitor System recorded the number of entries into the closed and open arms, the time spent in either type of the arms. After each trial the maze was wiped clean. Studied compounds and reference drug were administered (i.p.) 30 min before the test.

### Exploratory activity in rats

Exploratory activity in rats was measured using the EPM apparatus (described in section: Elevated plus maze test in rats), connected to a computer equipped with a program Motor Monitor System (Campden Instruments Ltd, UK). Each rat was gently placed in the centre of the plus-maze, facing one of the closed arms, immediately after a 5-min adaptation period in a plastic black box (60 cm x 60 cm x 35 cm). During a 5-min test period, the system recorded the total number of entries into arms and the total distance travelled. After each trial the maze was wiped clean. Studied compounds and reference drug were administered (i.p.) 30 min before the test.

### Data analysis

The data obtained were presented as mean ± S.E.M. and evaluated using one- or two-way analysis of variance (ANOVA), followed by Newman-Keuls or Bonferroni’s test *post hoc*, respectively. Differences between groups were considered significant if P<0.05. In a case of radioligand binding studies, the obtained data were fitted to a one-site curve-fitting equation with Prism 6.0 (GraphPad Software), and K_i_ values were estimated from the Cheng−Prusoff equation [36]:

$$K_{i}=\frac{{IC}_{50}}{1+\frac{L_{O}}{K_{D}}}$$

L_O−_labelled ligand concentration, K_D_−dissociation constant of labelled ligand

In a case of functional studies IC_50_ and EC_50_ were determined by nonlinear regression analysis using GraphPad Prism 5.0 software.

## Results

### The affinity of HBK-14 and HBK-15 for serotonergic 5-HT_2A_, SERT, adrenergic α_1_, α_2_ and β_1_ and dopaminergic D_2_ receptors

HBK-14 showed a high affinity for adrenergic α_1_ receptors, a moderate affinity for serotonergic 5-HT_2A_ and dopaminergic D_2_ receptors, and a very low affinity for SERT. It had no affinity for adrenergic α_2_ and β_1_ receptors (Table 1).

**Table 1:** The affinity for serotonergic, adrenergic and dopaminergic receptors and serotonin transporter (SERT).

Compound	Serotonergic K_i_ [nM]		Adrenergic K_i_ [nM]			Dopaminergic K_i_ [nM]
	5-HT_2A_	SERT	α_1_	α_2_	β_1_	D_2_
**HBK-14**	264	3500	23	n.a.	n.a.	219
**HBK-15**	109	529	13	n.a.	n.a.	54
**Mianserin**	2	—	—	—	—	—
**Escitalopram**	—	2	—	—	—	—
**Phentolamine**	—	—	9	—	—	—
**Clonidine**	—	—	—	3	—	—
**Propranolol**	—	—	—	—	8	—
**Haloperidol**	—	—	—	—	—	1

Inhibition constants (K_i_) were calculated according to the equation of Cheng and Prusoff [36].

n.a.–compound did not bind to receptor at 10^-5^M, SERT–serotonin transporter.

HBK-15 showed a high affinity for adrenergic α_1_ and dopaminergic D_2_ receptors, a moderate affinity for serotonergic 5-HT_2A_ and SERT, and no affinity for adrenergic α_2_ and β_1_ receptors (Table 1).

### The intrinsic activity of HBK-14 and HBK-15 towards serotonergic 5-HT_1A_ and 5-HT_7_ receptors

The intrinsic activity studies revealed activity of HBK-14 and HBK-15 at the target receptors and showed antagonist properties; however, their activity was weaker than that of reference antagonists, i.e. WAY-100635 (9-30-fold) and SB-269970 (51-147-fold) (Table 2).

**Table 2:** Functional data for the serotonergic 5-HT_1A_ and 5-HT_7_ receptors.

Receptor	Treatment	Agonist mode		Antagonist mode	
		E_max_ %	pEC_50_	IC_50_ nM	K_b_ nM
**5-HT**_**1A**_	**WAY-100635**	n.c.	n.c.	2.1	n.d.
	**Serotonin**	100	6.5	n.c.	n.c.
	**HBK-14**	6	n.c.	64	49
	**HBK-15**	8	n.c.	19	15
**5-HT**_**7**_	**Serotonin**	100	8.3	n.c.	n.c.
	**SB 269970**	4	n.c.	1.5	0.5
	**HBK-14**	19	n.c.	77	24
	**HBK-15**	0	n.c.	220	69

Data are expressed as the mean of two independent experiments in duplicate. E_max_—the maximum possible effect, pEC_50_—the logarithm of concentration of a compound where 50% of its maximal effect was observed; IC_50_ - (the half maximal inhibitory concentration) the concentration of a compound producing 50% inhibition of maximal effect; K_b_—the equilibrium dissociation constant of a competitive antagonist determined using of the Cheng-Prusoff equation [37]

n.c.—noncalculable

n.d.–not determined.

### Antidepressant-like activity of studied compounds in the FST in mice

**Fig 2:**
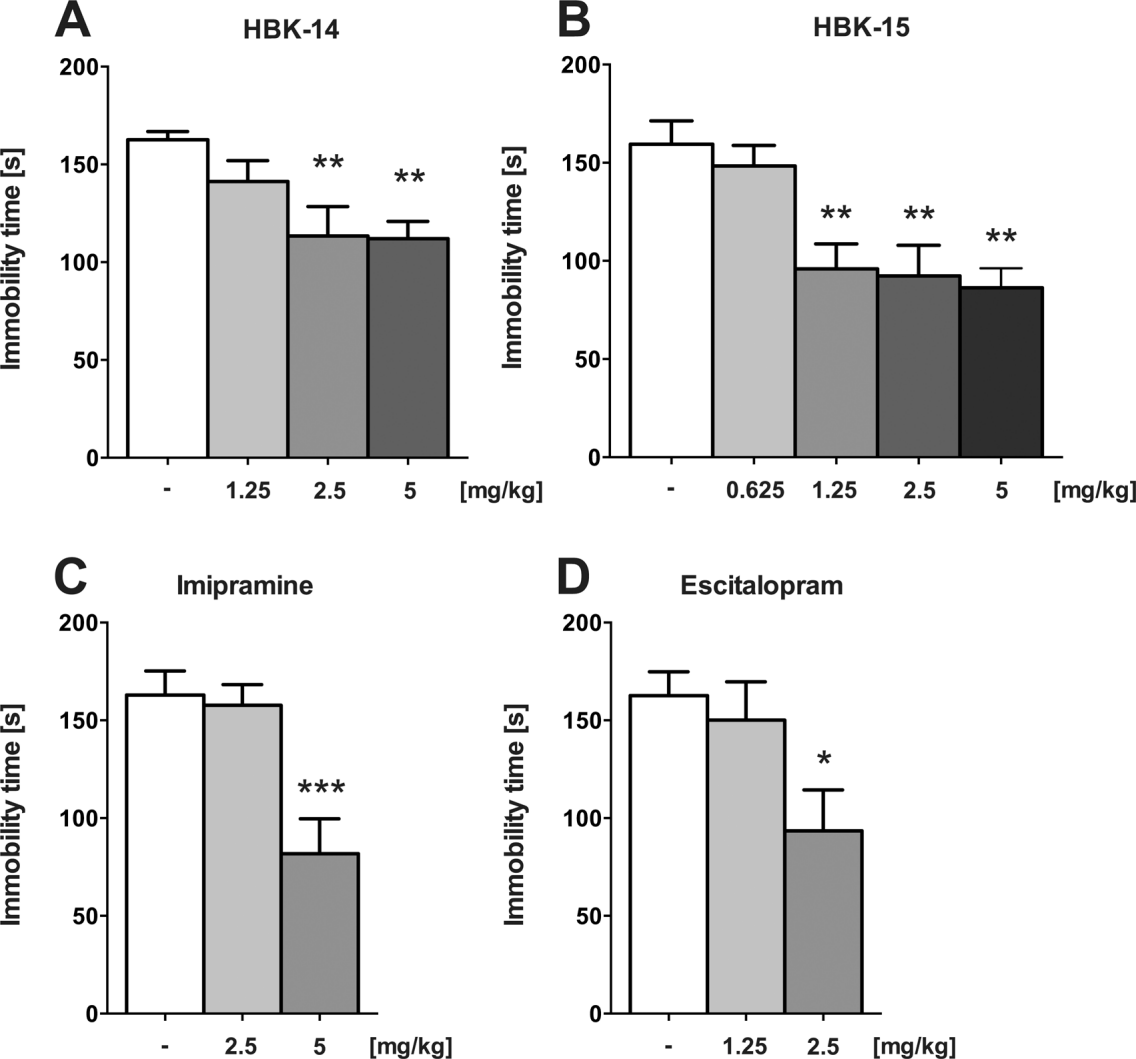
Antidepressant-like activity of HBK-14, HBK-15, imipramine and escitalopram in the FST in mice. HBK-14, HBK-15, imipramine and escitalopram were administered intraperitoneally 30 min before the test. The control groups received 0.9% NaCl. Statistical analysis: one-way ANOVA (Newman-Keuls *post hoc*) *P<0.05, **P<0.01, ***P<0.001, vs respective control group; n = 10 mice per group.

Fig 2A shows that HBK-14 (2.5 and 5 mg/kg) significantly decreased immobility time of mice (by 30% and 31%, respectively) [F(3,36) = 5.385, P = 0.0036]. HBK-15 (1.25, 2.5 and 5 mg/kg) significantly and dose-dependently reduced immobility time (by 40%, 42% and 46%, respectively) [F(4,45) = 7.897, P<0.0001] detected in FST in mice (Fig 2B). Imipramine (5 mg/kg) and escitalopram (2.5 mg/kg), given as reference drugs, decreased immobility in mice (by 50% [F(2,27) = 10.60, P = 0.0004] and 42% (F(2,27) = 4.177, P = 0.0263), respectively) (Fig 2C and 2D).

### Antidepressant-like activity of studied compounds in the FST in rats

**Fig 3:**
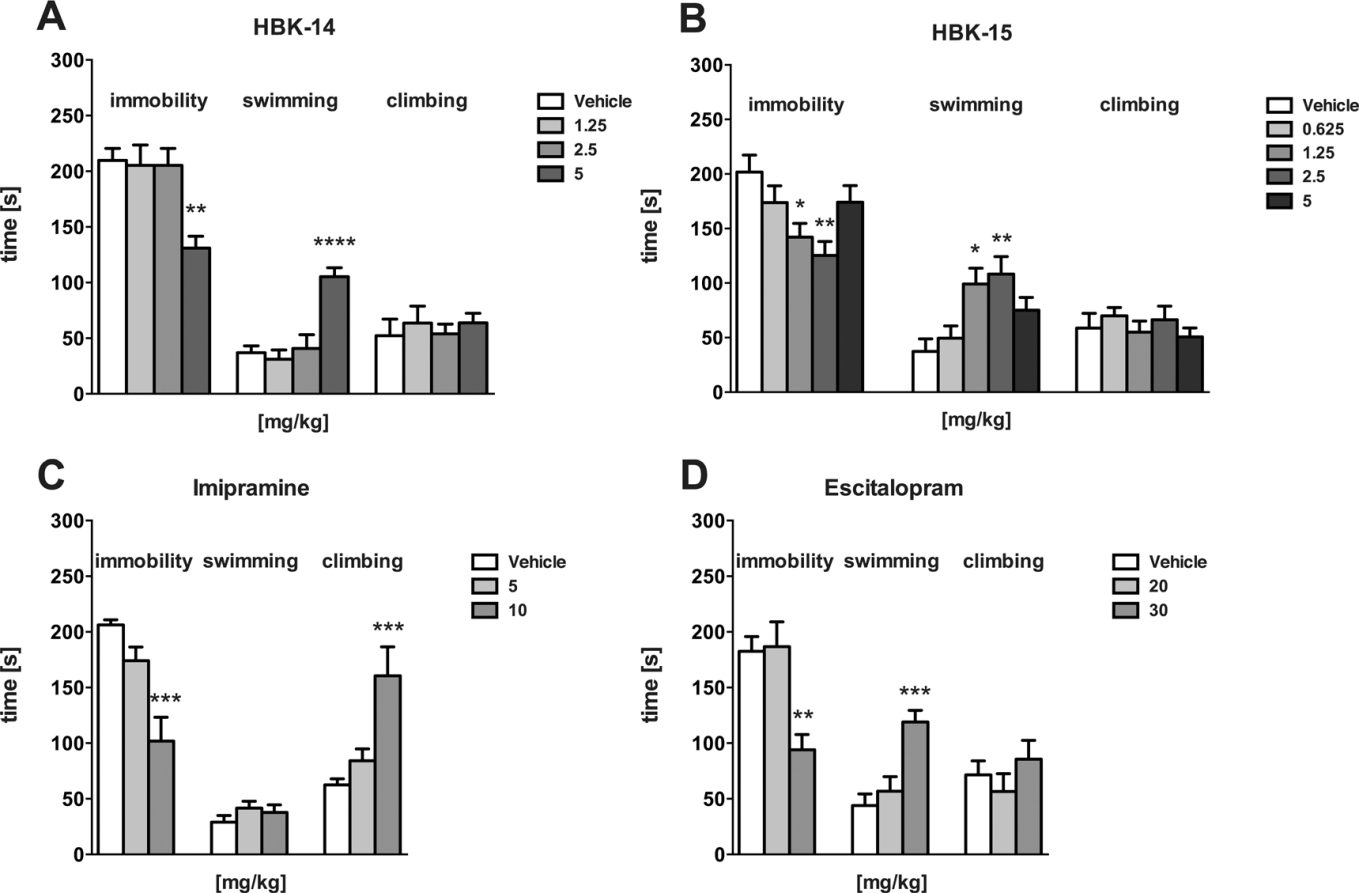
The effect on immobility, swimming and climbing behaviours of HBK-14, HBK-15, imipramine and escitalopram in the FST in rats. HBK-14, HBK-15, imipramine and escitalopram were administered intraperitoneally 30 min before the experiment. The control groups received 0.9% NaCl (vehicle). Statistical analysis: one-way ANOVA (Newman-Keuls *post hoc*) *P<0.05, **P<0.01, ***P<0.001 vs respective control group; n = 8 rats per group.

As demonstrated in Fig 3A, HBK-14 (5 mg/kg), compared with vehicle-treatment, significantly decreased immobility time (by 38%) [F(3,28) = 7.147, P = 0.0010], and increased swimming behaviour by 185% [F(3,28) = 4.146, P = 0.0010] in the rat FST. The compound showed no significant effect on the duration of climbing [F(3,28) = 2.439, P = 0.0854].

HBK-15 (1.25 and 2.5 mg/kg), compared with a control group, significantly decreased immobility time (by 30% and 38%, respectively) [F(4,35) = 4.375, P = 0.0057], and increased the time of swimming (by 166% and 191%, respectively) [F(4,35) = 5.422, P = 0.0017]. The tested compound had no significant influence on climbing [F(4,35) = 0.5547, P = 0.6969] in the FST in rats (Fig 3B).

Imipramine (10 mg/kg) significantly decreased immobility time (by 51%) [F(2,21) = 13.64, P = 0.0002] and increased climbing behaviour (by 163%) [F(2,21) = 9.872, P = 0.0009] in rats in the FST. The reference drug had no significant effect on the duration of swimming [F(2,21) = 1.056, P = 0.3656] (Fig 3C).

Escitalopram (30 mg/kg), compared with a control group, significantly decreased immobility time (by 48%) [F(2,21) = 9.516, P = 0.0011] and increased swimming (by 171%) [F(2,21) = 12.35 P = 0.0003], with no influence on climbing behaviour [F(2,21) = 1.008, P = 0.3818] of rats in FST (Fig 3D).

### The influence of HBK-14 and HBK-15 on locomotor activity in mice

Neither HBK-14 nor HBK-15 influenced locomotor activity of mice in 1-min session [F(3,36) = 0.2263, P = 0.8775, F(3,36) = 0.7642, P = 0.5216, respectively] and in 4-min session [F(3,36) = 1.374, P = 0.2663, F(4,45) = 0.7444, P = 0.5668, respectively] (Table 3).

**Table 3:** The influence of HBK-14 and HBK-15 on locomotor activity in 1-min and 4-min session in mice.

Treatment	Dose (mg/kg)	Number of crossings ± SEM					
		1 min			4 min		
**Vehicle**	-	104	±	6	416	±	57
**HBK-14**	1.25	93	±	10	488	±	58
	2.5	95	±	14	542	±	35
	5	98	±	9	424	±	48
**Vehicle**	-	98	±	11	510	±	62
**HBK-15**	0.625		-		531	±	52
	1.25	108	±	9	459	±	43
	2.5	98	±	10	411	±	68
	5	103	±	10	442	±	58

HBK-14 and HBK-15 were injected intraperitoneally 30 min before the test. Vehicle-treated groups received 0.9% NaCl. Statistical analysis: one-way ANOVA (Newman-Keuls *post hoc*); n = 10 mice per group.

### The influence on exploratory activity of studied compounds in rats

As shown in Table 4 there was no significant effect of HBK-14 (2.5 and 5mg/kg) on the total number of open-arm entries and distance travelled in the EPM apparatus [F(3,27) = 2.080, P = 0.1264, F (3,27) = 2.660, P = 0.0683, respectively]. HBK-15 at both doses had no effect on the parameters measured [F(2,19) = 0.0205, P = 0.9798 –the total number of arm entries, F(2,19) = 2.400, P = 0.1177 –total distance travelled]. Similarly, diazepam (1.25 and 2.5 mg/kg) did not influence the total number of arm entries and distance travelled by rats [F(2,22) = 0.4425, P = 0.6480, F(2,22) = 0.0425, P = 0.9585, respectively] (Table 4).

**Table 4:** Effects of HBK-14, HBK-15 and diazepam on exploratory activity in the elevated plus maze test in rats.

Treatment	Dose	Total arm entries ± SEM			Total distance travelled ± SEM (m)		
	(mg/kg)						
**Vehicle**	-	19.0	±	0.2	27.3	±	2.3
**HBK-14**	1.25	23.0	±	4.7	36.1	±	1.8
	2.5	20.7	±	5.1	30.0	±	5.8
	5	34.8	±	4.6	39.1	±	0.9
**Vehicle**	-	17.1	±	3.2	30.9	±	2.8
**HBK-15**	2.5	18.2	±	7.1	36.3	±	1.0
	5	16.8	±	3.8	30.9	±	2.2
**Vehicle**	-	11.3	±	2.9	28.5	±	2.7
**Diazepam**	1.25	15.8	±	4.1	29.1	±	3.1
	2.5	17.0	±	4.0	31.9	±	6.0

HBK-14, HBK-15 and diazepam were administered intraperitoneally 30 min before the test. Statistical analysis: one-way ANOVA (Newman–Keuls *post hoc*); n = 7–8 rats per group

### The effect of joint administration of sub-effective doses of studied compounds and fluoxetine, reboxetine or bupropion in the FST in mice

**Fig 4:**
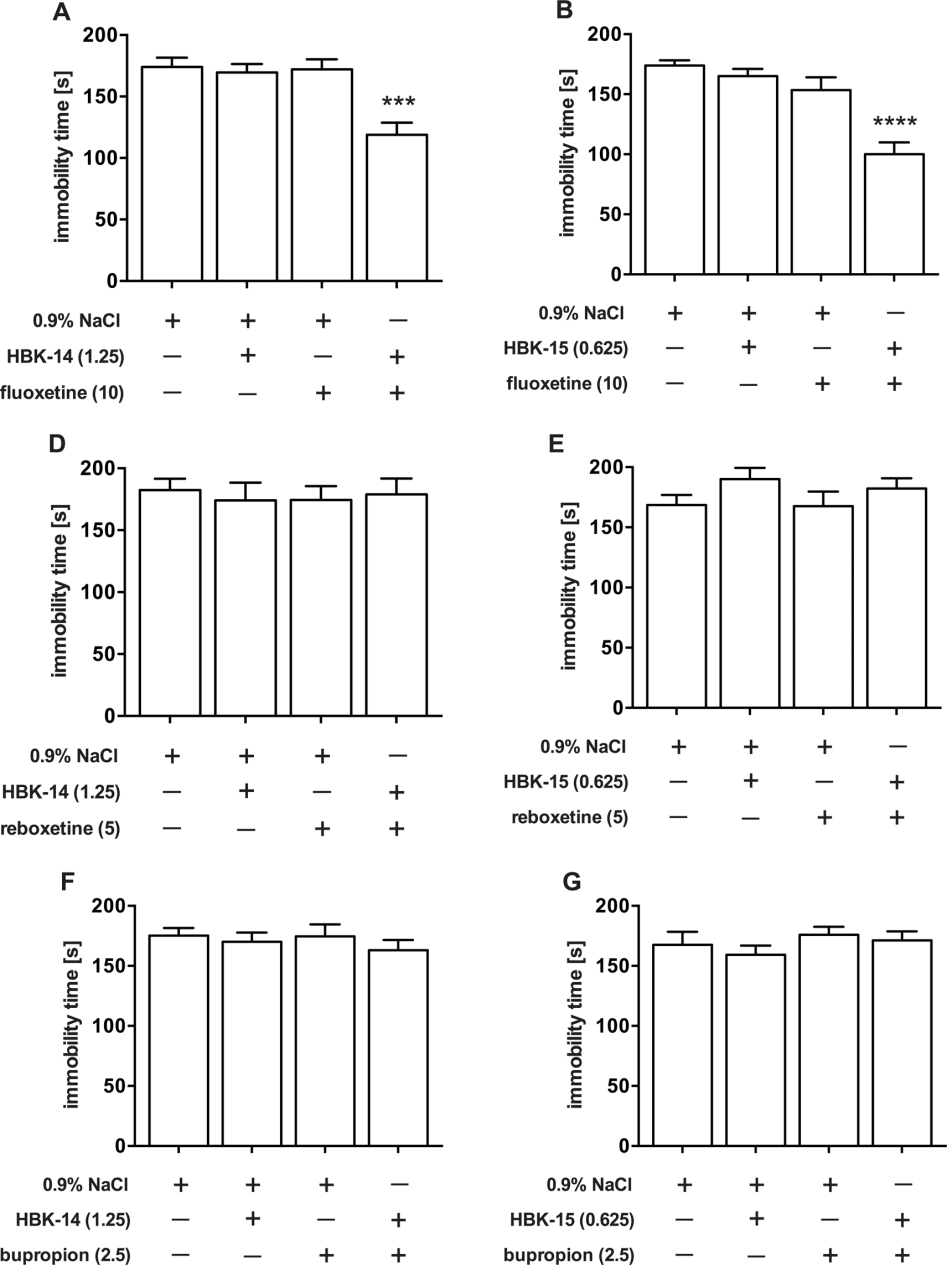
The effect of joint administration of sub-effective doses of studied compounds and fluoxetine, reboxetine or bupropion in the FST in mice. HBK-14, HBK-15, fluoxetine, reboxetine and bupropion were administered intraperitoneally 30 min before the test. The control groups received 0.9% NaCl. The doses (mg/kg) are indicated in brackets. Statistical analysis: two-way ANOVA (Bonferroni *post hoc*) ***P<0.001, ****P<0.0001 vs respective control group receiving 0.9% NaCl; n = 10 mice per group.

Fig 4 shows that all antidepressants given alone, i.e. fluoxetine (10 mg/kg), reboxetine (5 mg/kg) and bupropion (2.5 mg/kg) did not affect the immobility time of mice in FST. Similarly, none of tested compounds (HBK-14 1.25 mg/kg and HBK-15 0.625 mg/kg) administered alone affected the mouse immobility (Fig 4). The joint administration of HBK-14 (1.25 mg/kg) and fluoxetine (10 mg/kg) significantly decreased the immobility time (by 32%) (Fig 4A). Two-way ANOVA presents a significant interaction between HBK-14 and fluoxetine [F(1,36) = 8.905, P = 0.0051]. Similarly, HBK-15 (0.625 mg/kg) co-administered with fluoxetine (10 mg/kg) caused a significant reduction (by 43%) of immobility (Fig 4B). Two-way ANOVA presents a significant interaction between HBK-15 and fluoxetine [F(1,36) = 4.322, P = 0.0448].

Co-administration of HBK-14 (1.25 mg/kg) with reboxetine (5 mg/kg) had no effect on immobility time measured in the FST (Fig 4C). Two-way ANOVA demonstrates no interaction between HBK-14 and reboxetine [F(1,36) = 0.2838, P = 0.5975]. Similarly, the joint administration of HBK-15 (0.625 mg/kg) and reboxetine (5 mg/kg), as well as both compounds injected alone did not cause any changes in the immobility (Fig 4D). Two-way ANOVA demonstrates no interaction between HBK-15 and reboxetine [F(1,36) = 0.2511, P = 0.7328].

Combined administration of HBK-14 (1.25 mg/kg) and bupropion (2.5 mg/kg) did not influence the immobility time in the FST (Fig 4E). Two-way ANOVA shows no interaction between HBK-14 and bupropion [F(1,36) = 0.1551, P = 0.6960]. Similarly, HBK-15 (0.625 mg/kg) given jointly with bupropion (2.5 mg/kg) did not influence immobility time of mice (Fig 4F). Two-way ANOVA shows and no interaction between HBK-15 and bupropion [F(1,36) = 0.0497 P = 0. 8248].

### The effect of pretreatment with pCPA or WAY-100635 on antidepressant-like activity of studied compounds in the FST in mice

**Fig 5:**
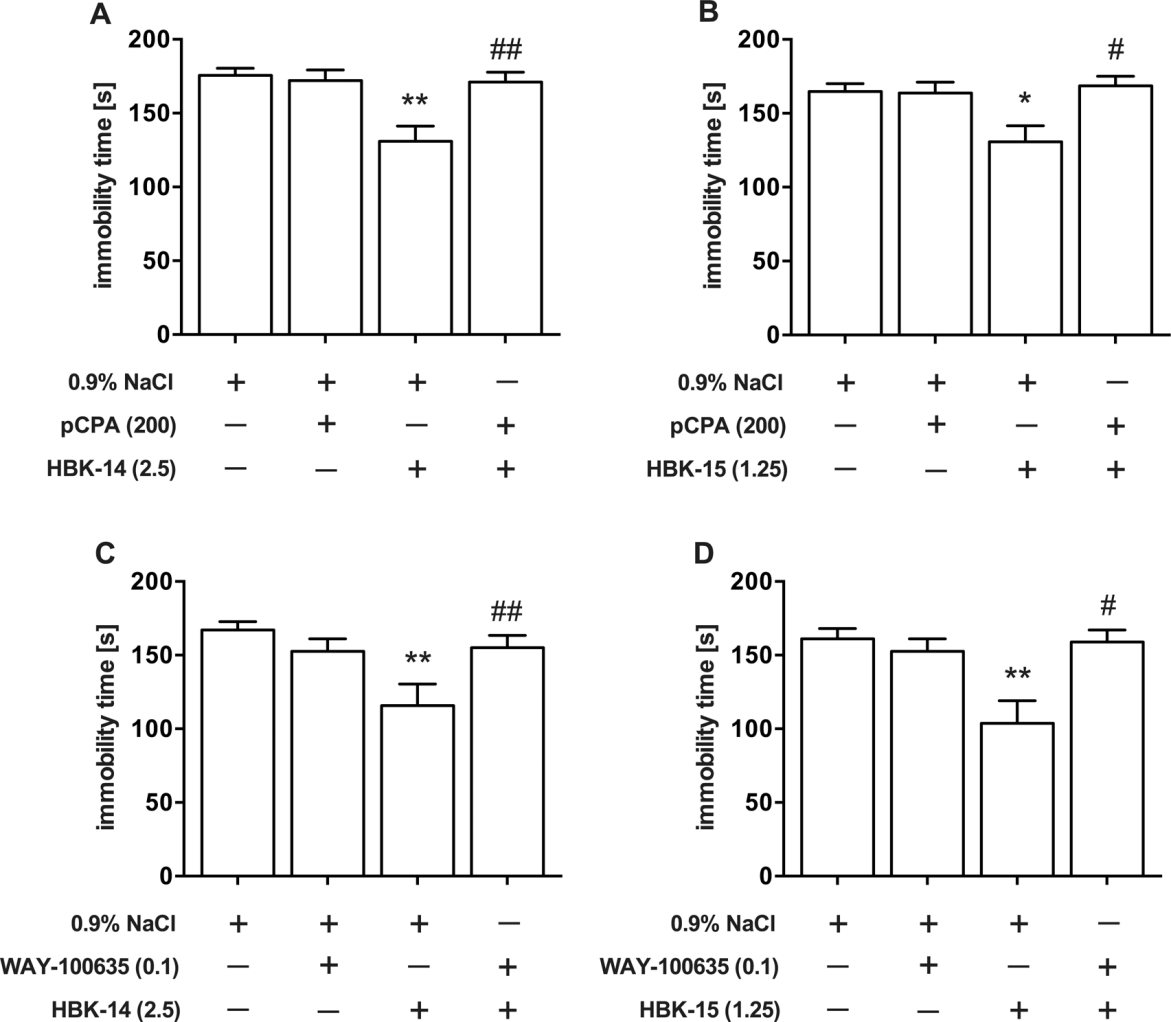
The effect of pretreatment with pCPA or WAY-100635 on antidepressant-like activity of HBK-14 and HBK-15 in the FST in mice. p-Chlorophenylalanine (pCPA) was injected intraperitoneally (i.p.) once daily for 3 consecutive days, and 24 h after the last injection and 30 min before the test mice received (i.p.) either the studied compound or 0.9% NaCl. WAY-100635 was injected subcutaneously 15 min before the studied compounds. The doses (mg/kg) are indicated in brackets. Statistical analysis: two-way ANOVA (Bonferroni *post hoc*) *P<0.05, **P<0.01 vs respective control group receiving 0.9% NaCl, #P<0.05, ##P<0.01 vs respective group receiving the studied compound; n = 10 mice per group.

HBK-14 (2.5 mg/kg) significantly reduced immobility (by 25%) in the FST in mice, whereas 3-day treatment with pCPA (200 mg/kg) did not influence immobility but antagonized the activity of HBK-14 in this test (Fig 5A). Two-way ANOVA demonstrates a significant interaction between HBK-14 and pCPA [F(1,36) = 8.312, P = 0.0066].

HBK-15 (1.25 mg/kg) significantly reduced immobility (by 22%) in the FST in mice, whereas 3-day treatment with pCPA (200 mg/kg) did not influence immobility time; however, it antagonized the antidepressant activity of HBK-15 in this test (Fig 5B). Two-way ANOVA demonstrates a significant interaction between HBK-15 and pCPA [F(1,36) = 7.208, P = 0.0109].

Administration of WAY-100635 (0.1 mg/kg) had no effect on the duration of immobility time by itself (Figs 5C and 4D). However, it completely antagonized the antidepressant effect elicited by HBK-14 in this test. Two-way ANOVA shows a significant interaction between HBK-14 and WAY-100635 [F(1,36) = 9.519, P = 0.0039]. Similarly, administration of WAY-100635 (0.1 mg/kg) completely antagonized the effect evoked by HBK-15 in FST. Two-way ANOVA shows a significant interaction between HBK-15 and WAY-100635 [F(1,36) = 7.443, P = 0.0098].

### The influence of joint administration of HBK-14 and HBK-15 with fluoxetine, bupropion, reboxetine, pCPA or WAY-100635 on locomotor activity in mice

The treatment with HBK-14, fluoxetine or their combination had no influence on locomotor activity of mice (Table 5). Two-way ANOVA shows no significant interaction between HBK-15 and fluoxetine [F(1,36) = 3.535, P = 0.0682]. The joint administration of HBK-15 and fluoxetine, as well as both compounds given alone, did not influence locomotor activity of mice (Table 5). Two-way ANOVA shows no significant interaction between HBK-15 and fluoxetine [F(1,36) = 0.1425, P = 0.7080].

**Table 5:** The effect of HBK-14 and HBK-15 given alone or in combination with fluoxetine, reboxetine or bupropion on locomotor activity in mice.

Treatment	Number of crossings ± SEM		
**Vehicle**	422	±	50
**HBK-14 (1.25)**	511	±	16
**fluoxetine (10)**	488	±	14
**HBK-14 (1.25) + fluoxetine (10)**	427	±	58
**Vehicle**	410	±	47
**HBK-15 (0.625)**	477	±	47
**fluoxetine (10)**	393	±	41
**HBK-15 (0.625) + fluoxetine (10)**	494	±	46
**Vehicle**	495	±	41
**HBK-14 (1.25)**	477	±	47
**reboxetine (5)**	393	±	41
**HBK-14 (1.25) + reboxetine (5)**	494	±	46
**Vehicle**	464	±	39
**HBK-15 (0.625)**	399	±	61
**reboxetine (5)**	366	±	37
**HBK-15 (0.625) + reboxetine (5)**	365	±	58
**Vehicle**	443	±	61
**HBK-14 (1.25)**	356	±	39
**bupropion (2.5)**	418	±	40
**HBK-14 (1.25) + bupropion (2.5)**	482	±	23
**Vehicle**	406	±	37
**HBK-15 (0.625)**	349	±	49
**bupropion (2.5)**	409	±	44
**HBK-15 (0.625) + bupropion (2.5)**	343	±	43

Fluoxetine, reboxetine, bupropion, HBK-14 and HBK-15 were administered intraperitoneally 30 min before the test. Vehicle-treated groups received 0.9% NaCl. The doses (mg/kg) are indicated in brackets. Statistical analysis: two-way ANOVA (Bonferroni *post hoc*); n = 10 mice per group.

The treatment with HBK-14, reboxetine or their combination had no influence on locomotor activity of mice (Table 5). Two-way ANOVA shows no significant interaction between HBK-14 and reboxetine [F(1,36) = 1.836, P = 0.1838]. The joint administration of HBK-15 and reboxetine, as well as both compounds given alone, did not influence locomotor activity of mice (Table 5). Two-way ANOVA shows no significant interaction between HBK-15 and reboxetine [F(1,36) = 0.4307, P = 0.5158].

The treatment with HBK-14, bupropion or their combination had no influence on locomotor activity of mice (Table 5). Two-way ANOVA shows no significant interaction between HBK-14 and bupropion [F(1,36) = 0.3536, P = 0.5558]. The joint administration of HBK-15 and bupropion, as well as both compounds given alone, did not influence locomotor activity of mice (Table 5). Two-way ANOVA shows no significant interaction between HBK-15 and bupropion [F(1,36) = 0.0103, P = 0.9195].

HBK-14 (2.5 mg/kg), pCPA (200 mg) and their combination did not influence locomotor activity of mice in 1 min session (Table 6). Two-way ANOVA shows no significant interaction between HBK-14 and pCPA [F(1,36) = 0.4090, P = 0.5265]. Analogously, neither HBK-14 (2.5 mg/kg), pCPA (200 mg) nor their combination influenced locomotor activity of mice in 4 min session (Table 6). Two-way ANOVA shows no significant interaction between HBK-14 and pCPA [F(1,36) = 0.3062, P = 0.5834].

**Table 6:** The effect of the pretreatment with pCPA or WAY-100635 on locomotor activity evoked by HBK-14 or HBK-15 in 1-min and 4-min session in mice.

Treatment	Number of crossings ± SEM					
	1 min			4 min		
**Vehicle**	92	±	11	400	±	38
**HBK-14 (2.5)**	105	±	12	344	±	46
**pCPA (200)**	99	±	11	370	±	27
**HBK-14 (2.5) + pCPA (200)**	98	±	10	350	±	44
**Vehicle**	103	±	13	380	±	42
**HBK-15 (2.5)**	93	±	14	356	±	41
**pCPA (200)**	88	±	11	408	±	20
**HBK-15 (2.5) + pCPA (200)**	88	±	10	391	±	30
**Vehicle**	102	±	8	423	±	38
**HBK-14 (2.5)**	99	±	9	363	±	42
**WAY-100635 (0.1)**	95	±	11	402	±	36
**HBK-14 (2.5) + WAY-100635 (0.1)**	101	±	6	460	±	55
**Vehicle**	117	±	8	434	±	58
**HBK-15 (2.5** ^a^ **/1.25** ^b^ **)**	99	±	7	360	±	49
**WAY-100635 (0.1)**	107	±	12	454	±	65
**HBK-15 (2.5** ^a^ **/1.25** ^b^ **) + WAY-100635 (0.1)**	100	±	9	441	±	50

p-Chlorophenylalanine (pCPA) was injected intraperitoneally (i.p.) once daily for 3 consecutive days, and 24 h after the last injection and 30 min before the test mice received (i.p.) either the studied compound or 0.9% NaCl. WAY-100635 was injected subcutaneously 15 min before the studied compounds. The doses (mg/kg) are indicated in brackets. Statistical analysis: two-way ANOVA (Bonferroni *post hoc*); n = 10 mice per group

^a^ 1-min session

^b^ 4-min session.

HBK-15 (2.5 mg/kg), pCPA (200 mg) or their combination did not influence locomotor activity of mice in 1 min session (Table 6). Two-way ANOVA shows no significant interaction between HBK-15 and pCPA [F(1,36) = 0.1899, P = 0.6656]. Similarly, HBK-15 (2.5 mg/kg), pCPA (200 mg) or their combination did not influence locomotor activity of mice in 4 min session (Table 6). Two-way ANOVA shows no significant interaction between HBK-15 and pCPA [F(1,36) = 0.0101, P = 0.9199].

The treatment with WAY-100635 (0.1 mg/kg), HBK-14 (2.5 mg/kg) and both compounds administered jointly did not influence locomotor activity of mice in 1-min session (Table 6). Two-way ANOVA shows no significant interaction between HBK-14 and WAY-100635 [F(1,36) = 0.3114, P = 0. 5803]. Similarly, the administration of WAY-100635 (0.1 mg/kg), HBK-14 (2.5 mg/kg) and both compounds jointly did not influence locomotor activity of mice in 4-min session (Table 6). Two-way ANOVA shows no significant interaction between HBK-14 and WAY-100635 [F(1,36) = 1.890, P = 0.1777].

The injection of WAY-100635 (0.1 mg/kg), HBK-15 (2.5 mg/kg) and both compounds jointly did not influence locomotor activity of mice in 1-min session (Table 6). Two-way ANOVA shows no significant interaction between HBK-15 and WAY-100635 [F(1,36) = 0.3208, P = 0.5746]. Similarly, the treatment with WAY-100635 (0.1 mg/kg), HBK-15 (1.25 mg/kg) and both compounds administered jointly did not influence locomotor activity of mice in 4-min session (Table 6). Two-way ANOVA shows no significant interaction between HBK-15 and WAY-100635 [F(1,36) = 0.3020, P = 0.5860].

### Anxiolytic-like activity of studied compounds in the four-plate test in mice

**Fig 6:**
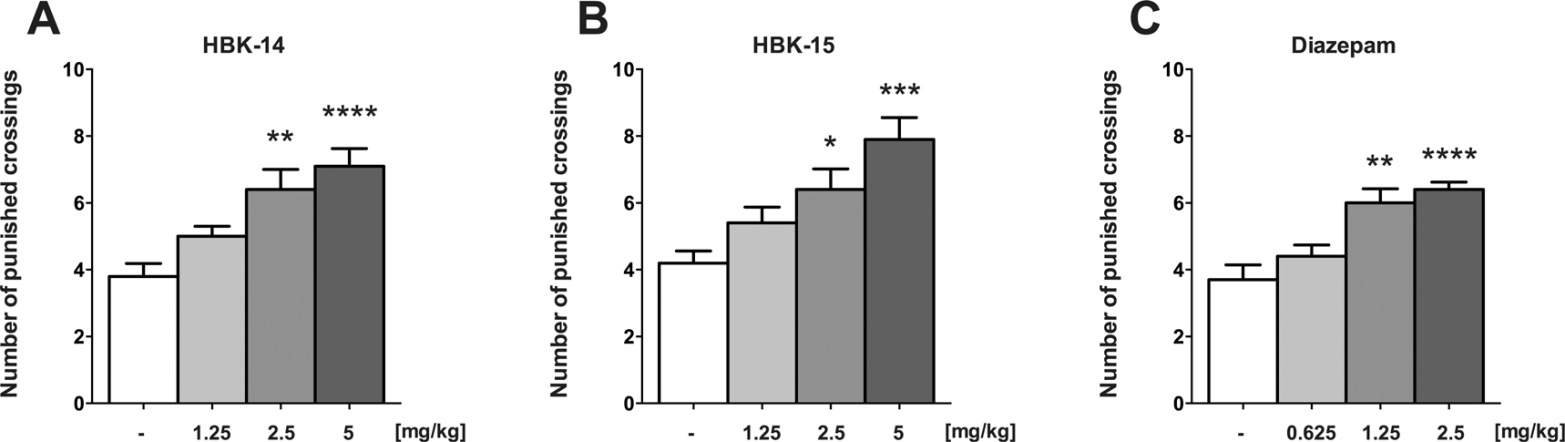
The anxiolytic-like activity of HBK-14, HBK-15 and diazepam in the four-plate test in mice. HBK-14, HBK-15 and diazepam were administered intraperitoneally 30 min before the experiment. The control groups received 0.9% NaCl. Statistical analysis: one-way ANOVA (Newman-Keuls *post hoc*) *P<0.05, **P<0.01, ***P<0.001, ****P<0.0001 vs respective control group; n = 10 mice per group.

Fig 6A shows that HBK-14 (2.5 and 5 mg/kg) significantly and dose-dependently increased the number of punished crossings (by 68% and 87%, respectively) [F(3,36) = 9.867, P<0.0001], in the four-plate test in mice. HBK-15 (2.5 and 5 mg/kg) also increased the parameter measured (by 52% and 88%, respectively) in that test acting in the dose-dependent manner (F(3,36) = 8.396, P = 0.0002) (Fig 6B). Diazepam (1.25 and 2.5 mg/kg), given as a reference anxiolytic, increased the number of punished crossings (by 62% and 73%, respectively) in the four-plate test in mice [F(3,36) = 12.14, P<0.0001] (Fig 6C).

### Anxiolytic-like activity of studied compounds in the EPM in rats

**Fig 7:**
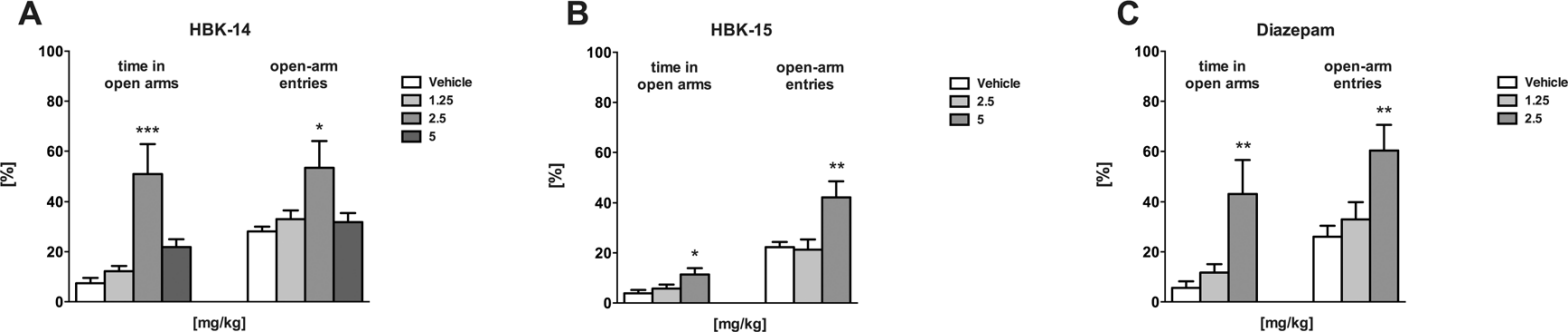
The influence of HBK-14, HBK-15 and diazepam on the time spent in open arms and open-arm entries in the elevated plus maze in rats. HBK-14, HBK-15 and diazepam were administered intraperitoneally 30 min before the experiment. The control group received 0.9% NaCl. Statistical analysis: one-way ANOVA (Newman-Keuls *post hoc*) *P<0.05, **P<0.01, ***P<0.001 vs respective control group; n = 7–8 rats per group.

As demonstrated in Fig 7A HBK-14 (2.5 mg/kg) significantly increased the percentage of time spent in the open arms (by 591%) [F(3,27) = 9.238, P = 0.0002], as well as the percentage of open-arm entries (by 90%) [F(3,27) = 3.543, P = 0.0277]. HBK-15 (5 mg/kg) showed a similar but weaker effect, increasing the percentage of time spent in the open arms (by 198%) [F(2,19) = 4.351, P = 0.027] and percentage of open-arm entries (by 89%) (F (2,19) = 6.692, P = 0.0063) (Fig 7B). Diazepam (2.5 mg/kg) significantly increased the percentage of both parameters measured in EPM, i.e. time spent in the open arms (by 672%) [F(2,20) = 8.474, P = 0.0022] and open-arm entries (by 131%) [F(2,20) = 6.249, P = 0.0078] (Fig 7C).

### The effect of pretreatment with pCPA or WAY-100635 on anxiolytic-like activity of studied compounds in the four-plate test in mice

**Fig 8:**
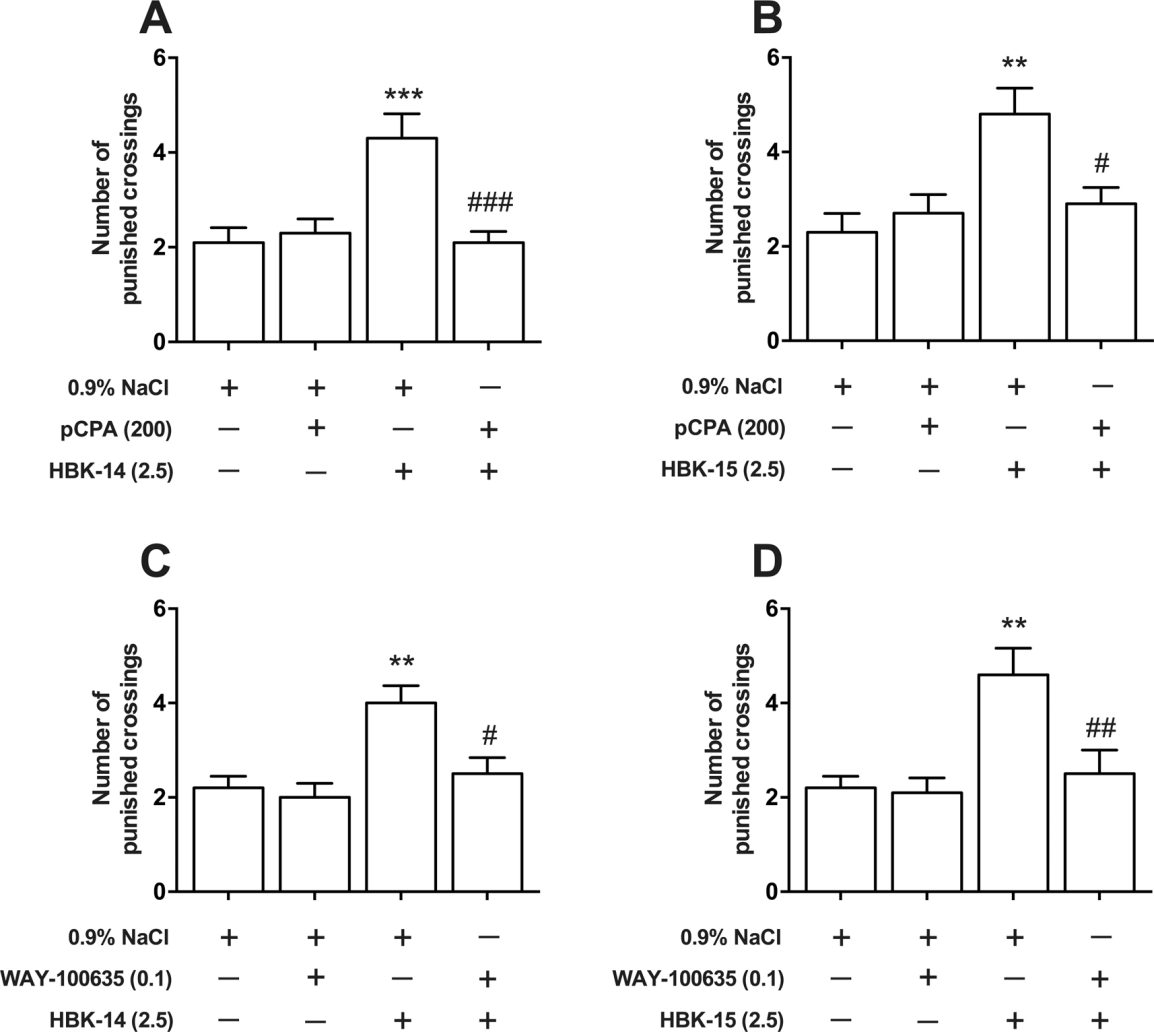
The effect of pretreatment with pCPA or WAY-100635 on anxiolytic-like activity of HBK-14 and HBK-15 in the four-plate test in mice. p-Chlorophenylalanine (pCPA) was injected intraperitoneally (i.p.) once daily for 3 consecutive days, and 24 h after the last injection and 30 min before the test mice received (i.p.) either the studied compound or 0.9% NaCl. WAY-100635 was injected subcutaneously 15 min before the studied compounds. The doses (mg/kg) are indicated in brackets. Statistical analysis: two-way ANOVA (Bonferroni *post hoc*) **P<0.01, ***P<0.001 vs respective control group receiving 0.9% NaCl, #P<0.05, ##P<0.01 vs respective group receiving the studied compound; n = 10 mice per group.

HBK-14 (2.5 mg/kg) increased the number of punished crossings (by 105%) in the four-plate test (Fig 8A). 3-day treatment with pCPA (200 mg/kg) had no effect on the number of punished crossings, but it completely antagonized the anxiolytic effect elicited by HBK-14 in this test. Two-way ANOVA shows a significant interaction between HBK-14 and pCPA [F(1,36) = 11.27, P = 0.0019].

Compared with vehicle treatment, HBK-15 (2.5 mg/kg) significantly increased (by 109%) the number of punished crossings in mice (Fig 8B). 3-day treatment with pCPA (200 mg/kg) showed no effect on the number of punished crossings in the four-plate test; however, it completely antagonized the effect evoked by HBK-15 in this test. Two-way ANOVA shows a significant interaction between HBK-15 and pCPA [F(1,36) = 7.138, P = 0.0113].

HBK-15 (2.5 mg/kg) increased the number of punished crossings (by 82%) in the four-plate test. Administration of WAY-100635 (0.1 mg/kg) had no effect on the number of punished crossings by itself, but it completely antagonized the effect elicited by HBK-14 in this test. Two-way ANOVA shows a significant interaction between HBK-14 and WAY-10635 [F(1,36) = 4.213, P = 0.0474] (Fig 8C).

Compared with vehicle treatment, HBK-15 (2.5 mg/kg) significantly increased (by 111%) the number of punished crossings in mice (Fig 8D). Administration of WAY-100635 (0.1 mg/kg) showed no effect on the number of punished crossings in the four-plate test; however, it completely antagonized the effect elicited by HBK-15 in this test. Two-way ANOVA shows a significant interaction between HBK-15 and WAY-100635 [F(1,36) = 5.505, P = 0.0246].

## Discussion

Our former studies revealed that HBK-14 and HBK-15, novel 2-methoxyphenylpiperazine derivatives, present a high affinity toward 5-HT_1A_ receptors (K_i_ = 41 nM and K_i_<1 nM, respectively), slightly weaker one toward 5-HT_7_ (K_i_ = 156 nM and K_i_ = 34 nM, respectively), and no affinity toward 5-HT_6_ receptors (K_i_ = 9967 nM and K_i_ = 9617 nM, respectively) [22]. As interesting dual 5-HT_1A_/5-HT_7_ ligands, the studies were continued and obtained findings have broadened their *in vitro* profile indicating a high interaction of HBK-14 and HBK-15 with α_1_-adrenoceptors and a weaker one with 5-HT_2A_ and D_2_ receptors. Furthermore, both compounds have low affinity for SERT and no affinity for adrenergic α_2_ and β_1_ receptors (Table 1). The results obtained in functional *in vitro* assays present both compounds tested as antagonists of serotonergic 5-HT_1A_ and 5-HT_7_ receptors; however, their activity did not reach as high antagonist level as that of reference WAY-100635 and SB-269970, respectively (Table 2).

It is well known that 5-HT_7_ receptor antagonists possess antidepressant- and anxiolytic-like properties present in various animal models [29,38]. Furthermore, in depressed individuals the blockade of 5-HT_1A_ autoreceptors may be beneficial, since in these patients an over-expression of presynaptic 5-HT_1A_ receptors has been reported [39].

For preliminary evaluation of antidepressant-like properties of studied compounds, we used the FST in mice. This test is a reliable tool for predicting the therapeutic potential of new compounds, in which a broad spectrum of antidepressants show activity, decreasing the time of behavioural despair of rodents [40]. We demonstrate that HBK-14 and HBK-15 produced dose-dependent antidepressant-like effects in that test, and the effect, in case of HBK-15, was even stronger than that of escitalopram and imipramine (Fig 2). None of the compounds tested affected locomotor activity of mice after administration at antidepressant-like doses (Table 3), thus we can conclude that observed antidepressant-like effects of all tested compounds are specific. Such antidepressant-like properties of HBK-14 and HBK-15 were also previously observed in mice in another screening test used, i.e. tail suspension test. So our present findings additionally endorse their antidepressant-like activity. In order to examine a possible mechanism of antidepressant-like action of the studied compounds in the FST in mice, we investigated their effect on the action of several antidepressants with diverse pharmacological profiles, administered at sub-effective doses. Interaction studies with conventional antidepressants are essential for conclusive assessment of antidepressant potential of a new compound [41], and may help to determine which system plays a role in the mechanism of its action [42]. Our studies revealed synergistic effect after joint administration of sub-effective doses of HBK-14 and HBK-15 with fluoxetine, but the combined treatment of both new compounds with reboxetine or bupropion did not cause any significant changes in immobility time duration (Fig 4). Since neither the studied compounds given alone nor their combinations with fluoxetine caused any changes in locomotor activity of mice (Table 3), the observed antidepressant-like effect was specific. Taking into account the above results, we can conclude that the antidepressant-like effects of HBK-14 and HBK-15 are most likely due to the interaction with the serotonergic system. To confirm its possible contribution in the antidepressant-like activity of the studied compounds, we depleted serotonin levels in mice by pretreatment with pCPA, a selective inhibitor of the rate-limiting enzyme in the biosynthesis of serotonin, tryptophan hydroxylase [43]. Studies have shown that the 3-day treatment with pCPA (200 mg/kg), significantly reduces the levels of serotonin in cortex, diencephalon, midbrain and pons & medulla in mice by around 30%, 35%, 24% and 34%, respectively [44]. The pretreatment with pCPA completely antagonised the antidepressant-like effect of HBK-14 and HBK-15 without changing the baseline activity of mice (Fig 5A and 5B, Table 6). This confirms that antidepressant-like activity of both studied compounds is conditioned by the integrity of serotonergic neurons. Such a concept is in line with data showing high affinity of both HBK-14 and HBK-15 toward 5-HT_1A_ receptors, which have been classified, as pre- and postsynaptic. The significance of 5-HT_1A_ receptor-mediated signalling in antidepressant-like effects observed in behavioural animal studies has been demonstrated [45]. To explore a role of 5-HT_1A_ receptors in the antidepressant-like effect of studied compounds, we pretreated mice with a selective 5HT_1A_ receptor antagonist—WAY-100635, since this compound, given alone, did not evoke any effect in the FST in mice (Fig 4C and 4D). Interestingly, the pretreatment with WAY-100635 significantly reduced antidepressant-like activity of both compounds in the FST (Fig 5C and 5D). As neither the studied compounds nor their combinations with WAY-100635 caused any changes in locomotor activity in mice, the observed antidepressant-like activity was specific (Table 6). Hence, it may be concluded that the activation of 5-HT_1A_ receptors is essential for the antidepressant-like effect of HBK-14 and HBK-15.

In the modified FST in rats both compounds showed antidepressant-like activity (Fig 3). The effect of HBK-14 was comparable to imipramine and stronger than escitalopram, and the activity of HBK-15 was stronger than the effect elicited by both studied reference drugs (Fig 3). It is notable that HBK-15 produced a U-shaped dose-response effect in rat FST. This effect is common for antidepressants with various mechanisms of action. It may be a result of some non-specific activity of the compound, which prevents a further decrease in immobility time [46]. Since none of the compounds influenced the exploratory activity of rats in the plus maze, the observed antidepressant-like activity was specific (Table 4). Similarly to escitalopram, both studied 2-methoxyphenylpiperazine derivatives significantly increased the time of swimming and did not influence climbing behaviours in the modified FST in rats (Fig 3). This pattern of rats' responding has been regarded as indicative of an enhanced serotonergic transmission and attributed to SSRIs action [33,47]. Thus, this increase in swimming yet not climbing behaviour indicates the serotonergic rather than the noradrenergic pathway's contribution to the antidepressant-like action of HBK-14 and HBK-15 in the FST in rats.

For preliminary evaluation of anxiolytic-like properties of investigated compounds, the four-plate test in mice was performed. This test is based on conflict situation, in which electric shock inhibits the mouse’s exploratory activity. Anxiolytics increase the number of punished crossings. Both HBK-14 and HBK-15 showed dose-dependent anxiolytic-like activity in the four-plate test in mice, but their effect was not as strong as the effect of diazepam (Fig 6). As neither HBK-14 nor HBK-15 affected locomotor activity in mice at anxiolytic doses (Table 3), the observed effect is be specific.

The function of serotonergic system, and especially 5-HT_1A_ receptors, is altered in patients with anxiety disorders [48]. Besides, 5-HT_1A_ receptors play a significant role in anxiolytic-like effects [49]. Taking it into account, we decided to examine the role of serotonergic system in mechanism of anxiolytic action of HBK-14 and HBK-15. In order to do that, we pretreated mice with pCPA for 3 consecutive days. The pretreatment with pCPA completely antagonised the anxiolytic-like effect of the studied compounds without changing the baseline activity of mice (Fig 8A and 8B, Table 6). This confirmed the integrity of serotonergic neurons in anxiolytic-like activity of HBK-14 and HBK-15. Moreover, the pretreatment with WAY-100635, which given alone did not evoke any effect in the mouse four-plate test, (Fig 7C and 7D), significantly reduced anxiolytic-like effects of both compounds in the four-plate test (Fig 8C and 8D). Since neither the studied compounds nor their combinations with WAY-100635 caused any changes in locomotor activity in mice, the observed anxiolytic-like activity was specific (Table 6). It may be concluded, though, that the activation of 5HT_1A_ receptors is essential for the appearance of anxiolytic-like effect of HBK-14 and HBK-15.

To confirm the anxiolytic-like properties of studied compounds, we performed EPM test. The test is based on rodents' aversion of open spaces, which results in avoiding of open areas by confining movements to enclosed spaces. Anxiolytics increase the open arm exploration time and open arm entries. In this model, both compounds produced an anxiolytic-like effect (Fig 7). We present that HBK-14 and HBK-15 increased the percentage of time spent in open arms and the percentage of open arms entries (Fig 7). The effect of HBK-14 was comparable to diazepam, and stronger than that of HBK-15. Our findings show that HBK-14 presents anxiolytic-like properties at one medium dose only. This is the common phenomenon with compounds with anxiolytic-like activity [50] and is difficult to explain on this stage of experimentation. As none of the compounds at anxiolytic-like doses affected the total number of arm visits and the distance travelled by rats, their anxiolytic-like action cannot be explained by competing behaviours, such as enhancement of locomotor activity (Table 4).

## Conclusions

In conclusion, in the present study we have demonstrated that HBK-14 and HBK-15 are weak serotonergic 5-HT_1A_ and 5-HT_7_ receptor antagonists, showing potent and specific antidepressant- and anxiolytic-like properties in various rodents’ models. Among the two, HBK-15 possesses stronger antidepressant-like activity both in mice and rats. In contrast, HBK-14 shows stronger anxiolytic-like activity especially in rats. Both compounds require intact serotonergic system and 5-HT_1A_ receptors’ activation, to display antidepressant- and anxiolytic-like effects. The promising results obtained so far should be corroborated by further studies conducted using other animal models to determine full pharmacological profile of the studied 2-methoxyphenylpiperazine derivatives.
